# Data on eosin Y solutions for laser-induced fluorescence in water flows

**DOI:** 10.1016/j.dib.2020.105350

**Published:** 2020-03-05

**Authors:** Luc Biasiori-Poulanges, Sébastien Jarny, Hazem El-Rabii

**Affiliations:** Institut Pprime, CNRS UPR 3346 - Université de Poitiers - ISAE-ENSMA, 1 avenue Clément Ader, 86961 Futuroscope, France

**Keywords:** Dye-water solution, Surface tension, Viscosity, pH, Laser-induced fluorescence

## Abstract

Dye tracing techniques involve the tagging of a sample of water with dye, providing important qualitative and quantitative information. This article presents physical and fluorescence properties of dye solutions obtained by diluting a pharmaceutical aqueous solution of eosin Y with distilled water. Sample solutions with eosin concentrations ranging from 0 to 20 g/L were examined under various temperatures and laser powers. The data include measurements of dynamic viscosity, surface tension and pH. Fluorescence emission spectra as well as laser beam attenuation and photobleaching measurements are also reported. The datasets provide guidelines for obtaining optimal dye mixtures and suitable optical configurations to implement eosin fluorescence techniques.

**Table:** Specifications Table

Subject	Fluid Flow and Transfer Processes.
Specific subject area	Multiphase flows and optical techniques for water flow visualization.
Type of data	Tables Graphs Figures
How data were acquired	Data include dynamic viscosities, surface tension, pH and fluorescence properties of eosin yellowish aqueous solutions. The dynamic viscosity measurements were carried out using a rotational rheometer D-HR2 (TA Instruments). Surface tension were measured by a drop shape analyzer DSA25 with the pendant drop method (Krüss). Measurement of pH were performed using a CG 820 microprocessor-based pH-Meter (Schott Geräte), a 90450 pH electrode (Bioblock) and a thermocouple type K (NiCr-Ni, 1/2 DIN IEC 584 class 2). Fluorescence spectra, from 500–750 nm under an excitation wavelength at 532 nm (Verdi-V5, Coherent), were acquired on a Ocean Optics USB2000+XR1-ES spectrometer. The loss of fluorescence by photobleaching and extinction coefficients were measured using a high-speed Photron Fastcam Mini AX50 camera.
Data format	Raw.
Parameters for data collection	Data include dynamic viscosities, surface tension, pH, extinction coefficients, and fluorescence properties of eosin yellowish aqueous solution at various controlled temperatures and concentrations. Fluorescence properties and extinction coefficients were measured at a specific laser-excitation power, while the loss of fluorescence by photobleaching was measured at several laser-excitation powers.
Description of data collection	Data were collected from aqueous solutions obtained by adding dropwise an eosin based pharmaceutical solution (disodium eosin 2.00 g, chlorphenesine, pentylene glycol, water 100 mL) into 100 mL of distilled water under magnetic stirrer. For the fluorescence and absorbance measurements, the dye solutions were contained within a quartz glass cuvette and horizontally illuminated by a laser sheet close to the glass wall.
Data source location	Institut Pprime, Chasseneuil-du-Poitou, France.
Data accessibility	Data are provided with the article.

**Table:** 

**Value of the Data** • The datasets characterize the fluorescent properties of Eosin Y that was supplied as a component of a pharmaceutical aqueous solution. As such, this dye may be a low-toxic alternative to toxic fluorescent tracers commonly used in laboratory fluid experiments. • The data are relevant to researchers investigating water and multiphase flows by means of fluorescence imaging techniques, as well as to researchers developing fluorescence models. • The data enable to determine the operating parameters required to implement fluorescent techniques. Specifically, they provide guidelines for obtaining optimal dye mixtures and suitable optical configurations (including laser fluence) for given experimental conditions. These guidelines may be based on the measurements of laser beam attenuation and the rate of the fluorescence signal loss due to photobleaching. • Beside the fluorescence properties of Eosin Y reported in the article, which provide an add value to the existing scientific literature, the data allow to assess to what extent the addition of Eosin Y alters some physical properties of water, in particular its viscosity and surface tension.

## 1 Data description

Laser-induced fluorescence (LIF) is a non-intrusive optical technique that uses natural or added tracers for flow visualization or quantitative measurements (e.g., temperature, concentration, pH, velocity) in many environments. LIF is commonly used in laboratory flow fields, with applications in fluid dynamics research, as well as in process and biomedical engineering [[1], [2], [3], [4], [5]]. Among the dyes with high quantum yield in water, disodium eosin (EY) is a low-toxic alternative (see Ref. [6] for a detailed discussion on the toxicity) to popular toxic fluorescent dyes used in laboratory flow experiments such as Rhodamine derivatives (e.g. Rhodamine6G, Rhodamine 110) [7]. It is water soluble and has a quantum yield in water of 0.36, with a sufficiently large Stokes shift [7,8], making it well-suited for use as a water tracer.

The datasets reported here relate to physical and fluorescence properties of EY dissolved in distilled water. More specifically, they concern EY that is commercially available as a pharmaceutical aqueous solution (EPAS) for cutaneous application. The composition of this solution is (according to the manufacturer, Cooper) 2 g of EY for 100 mL of purified water, with unspecified portion of chlorphenesine and pentylene glycol. Data include dynamic viscosities, surface tension, pH, extinction coefficients, and fluorescence signal measurements for EY aqueous solution at various controlled temperatures and concentrations. They allow to assess the following points:

- to what extent the addition of EY alters the viscosity and surface tension of water?
- is EY (excitated at 532 nm) capable of producing a strong enough fluorescence signal for reliable detection at low concentrations?
- what is the effect of the EY concentration on the attenuation of the excitation laser?
- to what extent prolonged exposure to light can destroy the ability of EY molecule to fluoresce?
- how does the gas-phase fluorescence signal of EY compare with that of the liquid phase? Does the LIF signal from the much denser liquid phase overwhelm the signal from the vapor phase?

**Fig. 1:**
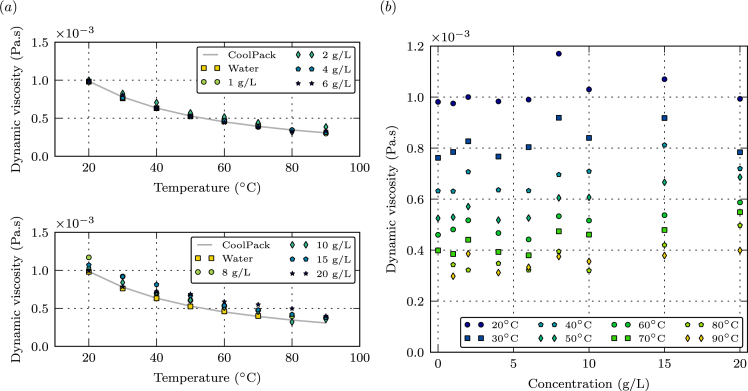
(a) Dynamic viscosity as a function of temperature. (b) Dynamic viscosity as a function of EY concentration.

**Fig. 2:**
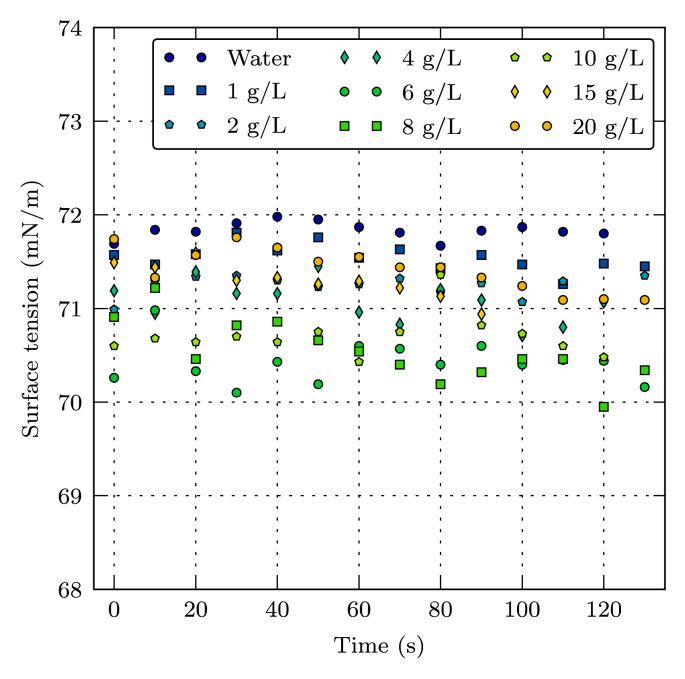
Surface tension as a function of time for various EY concentrations at 20 °C.

**Fig. 3:**
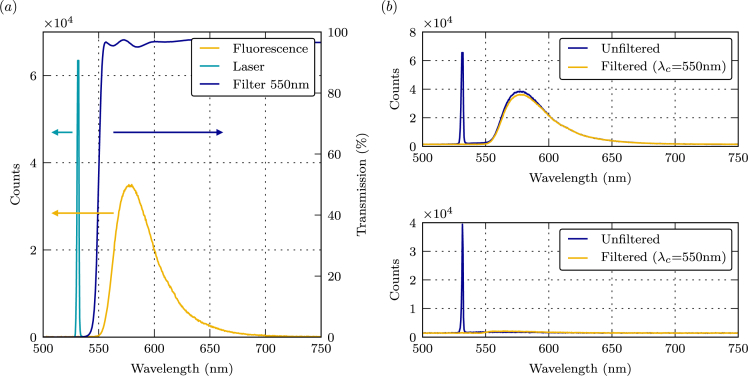
Emission spectra of EY fluorescent dyes, laser and bandwidth of the long-pass filter. The dark blue curve labeled ‘Filter 550 nm’ corresponds to the right ordinate axis scale. (b) Emission spectra in the liquid phase (top) and the gaseous phase (bottom).

**Fig. 4:**
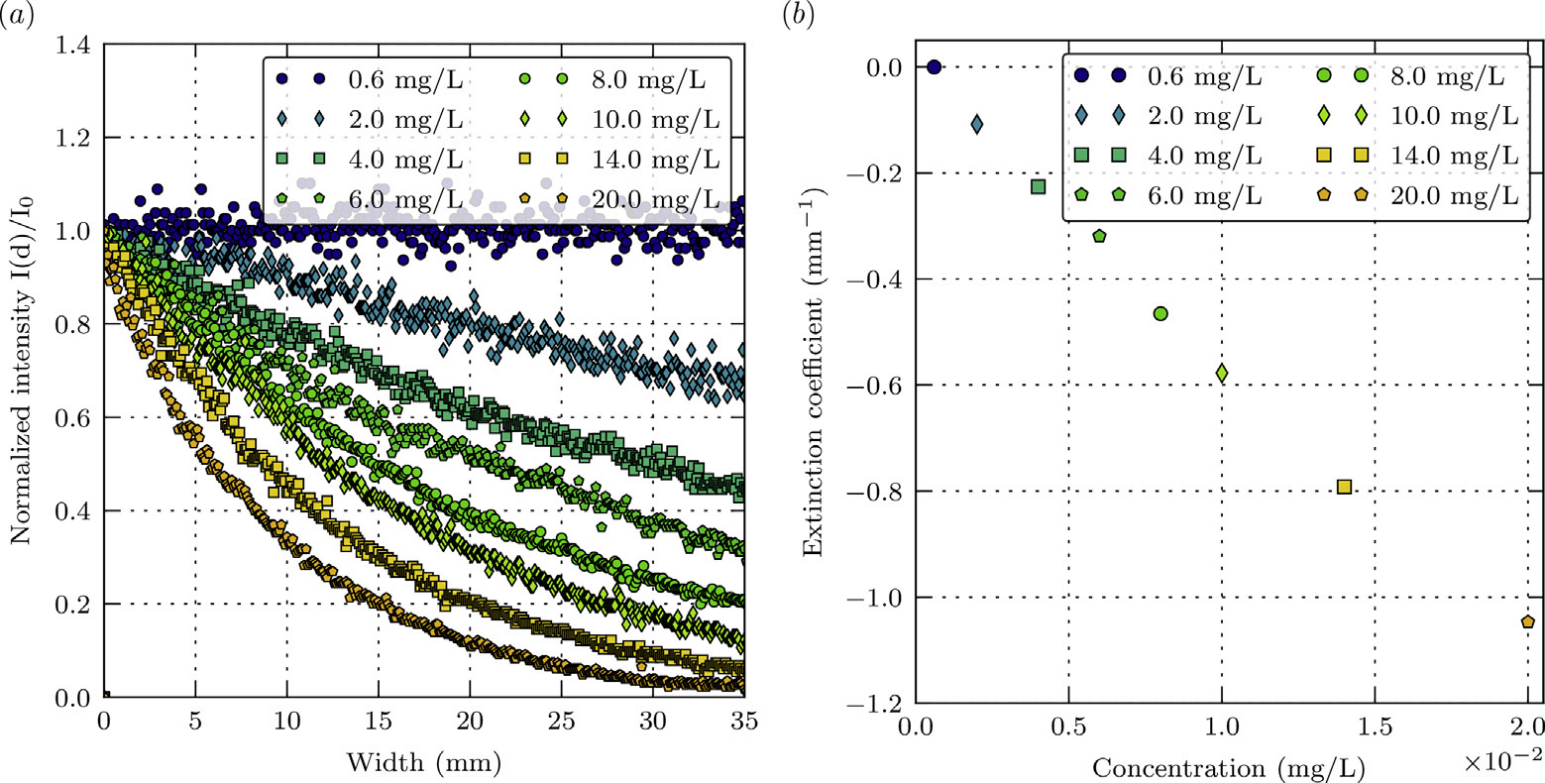
(a) Absorption as a function of the sample width. (b) Extinction coefficient as a function of the EY concentration.

**Fig. 5:**
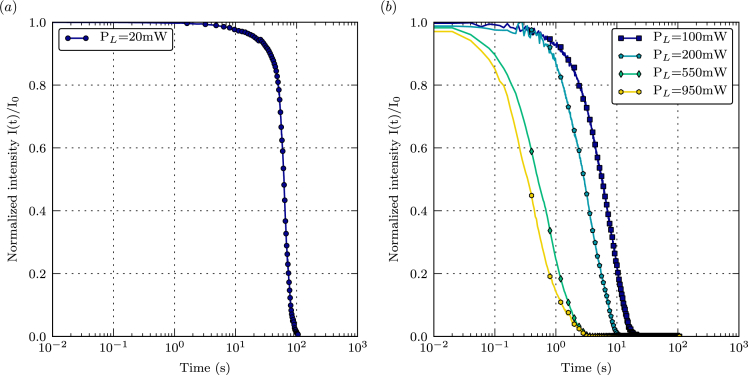
(a) Photobleaching as a function of time for a laser power output of 20 mW (smoothed with the Savitzky-Golay filter). (b) Photobleaching as a function of time for laser power output from 100 mW to 950 mW.

Table and graphic display of the data are shown in Table 1, Table 2, Table 3, Table 4 and Fig. 1, Fig. 2, Fig. 3, Fig. 4, Fig. 5. All concentrations reported in this paper are mass concentrations (i.e., mass of EY per volume of water).

Fig. 1(a) shows the changes in dynamic viscosity with temperature at different solution concentrations of EY. Dynamic viscosity versus EY concentration is given in Fig. 1(b). Temperature ranges from 20 °C to 90 °C (±0.25 °C), and EY concentration is from 0 to 20 g/L.

The measurements of surface tension at EY concentration from 0 to 20 g/L are presented in Fig. 2. The measurements were conducted at a temperature of 20 °C (±0.5 °C). This figure shows, for each concentration, the measurement of surface tension of a drop over a time period of 130 s, with a measurement every 10 s. Table 1 lists, for each concentration, the pendant drop volume, the mean surface tension value calculated from measurement points shown in Fig. 2, and the corresponding standard deviation.

**Table 1:** Mean, standard deviation and initial volume of the pendant drop.

C_EY_ (g/L)	Mean (mN/m)	Standard deviation (mN/m)	Initial volume (μL)
0.0	71.8	0.1	8.7
1.0	71.6	0.1	7.3
2.0	71.2	0.1	7.3
4.0	71.1	0.2	7.5
6.0	70.4	0.2	7.8
8.0	70.6	0.3	7.9
10.0	70.7	0.2	7.9
15.0	71.3	0.2	6.9
20.0	71.4	0.2	8.7

Table 2 reports measurements of solutions pH at EY concentration from 0 to 20 mg/L and the corresponding solution temperatures (±0.1 °C) during measurement.

**Table 2:** pH as a function of EY concentration.

C_EY_ (mg/L)	pH	Temperature (°C)
0.0	8.84	19.1
0.6	8.72	18.8
2.0	8.23	18.8
4.0	8.18	18.8
6.0	7.85	18.7
8.0	7.63	18.8
10.0	7.52	18.8
14.0	7.37	18.8
20.0	7.36	18.7

Fig. 3(a) shows the fluorescence emission spectra of a 200.0 mg/L EY solution measured at 20 °C (±0.5 °C) that was excited with green light (532 nm, 1 W). The emission spectra ranges from 550 to 700 nm with a peak at 578 nm. The excitation radiation and the transmission curve of the long-pass filter (550 nm cut-off wavelength) used to block the excitation light are also displayed. Fig. 3(b) shows the emission spectra emanating from the liquid and vapor phases. In contrast to the liquid phase, the vapor phase produces no detectable fluorescence signal within the wavelength range investigated.

Attenuation of the excitation laser beam against liquid width at various concentration (0.6–20.0 mg/L) are presented in Fig. 4(a). The measurements were conducted at 20 °C (±0.5 °C) and laser power output of 1 W. Fig. 4(b) shows the extinction coefficient (measured at 532 nm) versus concentration, under the same experimental conditions. Fig. 5(a) and (b) display the photobleaching of a 20.0 mg/L EY solution as a function of time at several laser powers (20–950 mW). Data on the photobleaching at 20 mW are smoothed to remove noise with the Savitzky-Golay filter, which is a moving unweighted linear least-squares regression with a second order polynomial approximation. For each concentration, Table 3 lists the maximal sample width over which fluorescence signal is reduced by 20%. Table 4 gives time elapsed before photobleaching reduces the fluorescence signal by 20%, for a 20.0 mg/L EY solution and at laser power from 20 to 950 mW. The measurements in Table 3, Table 4 were obtained at 20 °C (±0.5 °C).

**Table 3:** Maximal sample width as a function of EY concentration.

C_EY_ (mg/L)	Maximal sample width (mm)
0.6	>35.0
2.0	21.3
4.0	9.5
6.0	6.5
8.0	5.7
10.0	4.1
14.0	2.8
20.0	1.7

**Table 4:** Maximal time duration as a function of laser power output.

Laser power output (mW)	Maximal time duration (s)
20	50.0
100	2.40
200	1.30
550	0.24
950	0.18

## 2 Experimental design, materials, and methods

### 2.1 Sample preparation

Solution samples were prepared by diluting various amounts of EPAS with 100 mL of distilled water. EPAS was added dropwise into the distilled water under magnetic stirrer using a micrometer syringe. To prevent premature photodegradation, the prepared solutions were stored and protected from light exposure until analysis.

### 2.2 Dynamic viscosity

**Fig. 6:**
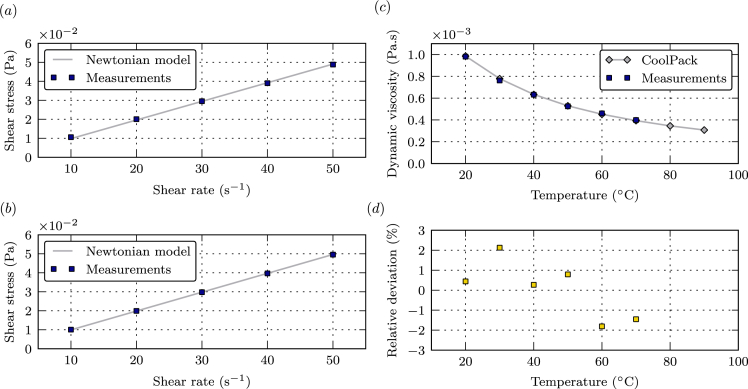
Rheograms and Newtonian laws for pure water at 20 °C (a) and pure eosin at 20 °C (b). CoolPack-experiments comparison of the dynamic viscosity of water at 20 °C (c). Relative deviation between CoolPack and experiments (d).

The viscosity measurements were obtained by the rotational method using a rotational rheometer with a double gap geometry (D-HR2, TA Instruments). All measurements were conducted following the same procedure. The liquid sample was first poured into the test cell. The temperature was set to a desired value (±0.25 °C), allowing a period of 30 min for thermal equilibrium before starting the measurement. During this period of time, a constant shear rate of 50 s^−1^ was applied to ensure temperature homogeneity within the sample. The shear stress measurements were then conducted for five shear-rate values from 50 to 10 s^−1^. At each measurement point, the shear rate was maintained until equilibrium of the torque was reached. As examples, the rheograms (shear stress vs. shear rate) of pure water and a 20 g/L EY solution at 20 °C are presented in Fig. 6(a) and (b). The dynamic viscosity was finally determined by fitting the rheogram data to the Newtonian fluid model $\tau =\mu \dot{\gamma }$, where τ is the shear stress, $\dot{\gamma }$ is the shear rate, and μ is the dynamic viscosity. The device calibration was checked by comparing the dynamic viscosity measurements for pure water, at 20 °C, to the data from the CoolPack software [9] [see Fig. 6(c) and (d)].

### 2.3 Surface tension

The surface tension of EY solution was measured at different concentrations by the pendant drop method using a drop shape analyser (DSA25, Krüss GmbH). A pendant drop was mechanically generated at the tip of a precision needle in air. A software-controlled dosing system, with a resolution of 0.1 μL and a speed of 10 μL/min, was used for the dosing operation. The drop interface was captured by imaging the drop using backlighting. The drop was imaged by means of a digital camera (CF03, Krüss GmbH) (1200 × 1200 pixels) equipped with a 6.5× magnification zoom lens, which provides a 2.8 μm/px resolution. Surface tension was then determined in a two-step process. First, the experimental images were processed by applying an edge detection algorithm by means of grey scale analysis to extract the drop profile. Second, the Young-Laplace equation was iteratively solved to find the geometrical and physical parameters that most precisely fit the drop profile using an optimisation technique. The accuracy of the measurement method is 0.3 mN/m.

### 2.4 pH measurements

The solutions pH were measured with a glass 90450 pH electrode (Bioblock) connected to a CG 820 pH-meter (Schott-Geräte, GmbH). The pH values were determined by insertion of the probe 30 mm into the solutions until a stable value was reached. A thermocouple type K NiCr-Ni (1/2 DIN IEC 584 class 2, ±0.1 °C) was added to allow for manual temperature compensation. Prior to each measurement, the electrode was rinsed with distilled water to avoid crossed-contamination of the solution and the pH-meter was calibrated against standard buffer solution at pH 7.01 (Fisherbrand). The accuracy of the pH-meter was ±0.01 pH unit.

### 2.5 Fluorescence emission spectra, laser beam attenuation and photobleaching

**Fig. 7:**
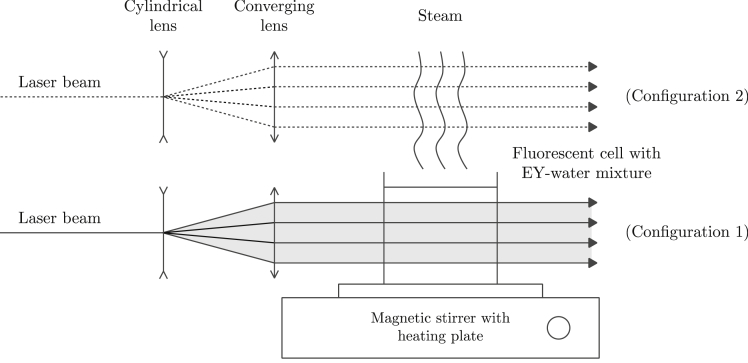
Planar Laser-Induced Fluorescence experimental setup in front view.

The experimental setup used to perform the laser beam attenuation and fluorescence measurements is shown in Fig. 7. The EY-water mixture investigated was contained within a 40-mm cubic quartz fluorescence cell that was placed on a magnetic hot plate stirrer with temperature controller. Fluorescence excitation was produced by a Coherent Verdi V5 Nd:YVO4 laser, which can provide up to 5 W of continuous wave (cw) radiation at a wavelength of 532 nm. A laser sheet was formed by diverging the beam in one dimension with a negative cylindrical lense (−10 mm) and focusing the beam in the perpendicular direction with a 200 mm spherical lens to produce a laser sheet approximately 200 μm thick. The laser sheet passed through the cell from left to right (Fig. 7, configuration 1). The fluorescence signal was then imaged, perpendicular to the laser sheet, using a Photron Fastcam AX-50 high-speed camera with a 1024 × 1024 pixel resolution, which was equipped with a 105-mm f/2.8 Sigma macro lens and a long-pass filter (cut-off wavelength of 550 nm). Laser beam attenuation was obtained from the fluorescence images by measuring the signal intensity along the cell width. By recording a series of images (frame rate 50 Hz, exposure time 20 ms), the development of photobleaching was observed from which the rate of fluorescence loss can be deduced.

Fluorescence emission spectra over 500–750 nm were captured by a USB2000+XR1-ES spectrometer (Ocean Optics). The scattered and fluorescence light were collected at right angle to the laser sheet through a 1000 μm core diameter optical fiber. The spectrometer resolution was between 1.7 nm and 2.1 nm (full width at half maximum). Scattered laser light was removed by placing the long-pass filter in front of optical fiber. Vapor fluorescence spectra were measured by passing the laser sheet horizontally only through the vapor phase approximately 1 mm above the liquid surface (Fig. 7, configuration 2). The fluorescence spectra from the vapor and liquid phase were measured in turns.
